# An observational study of immigrant mortality differences in Norway by reason for migration, length of stay and characteristics of sending countries

**DOI:** 10.1186/s12889-018-5435-4

**Published:** 2018-04-17

**Authors:** Astri Syse, Minja T. Dzamarija, Bernadette N. Kumar, Esperanza Diaz

**Affiliations:** 1 Statistics Norway, Oslo, Norway; 2 The Norwegian Centre for Minority Health Research, Norwegian Institute of Public Health, Oslo, Norway; 3 Department of Global Public Health and Primary Care, University of Bergen, Bergen, Norway

**Keywords:** Acculturation, Immigrant, Healthy migrant, Length of stay, Mortality, Norway, Reason for migration, Social causation

## Abstract

**Background:**

Knowledge of mortality differentials in immigrant groups depending on their reason for migration, length of stay in host countries and characteristics of sending countries may be beneficial for policy interventions aimed to improve various immigrant groups’ health and welfare.

**Methods:**

We employed discrete-time hazard regression models with time-varying covariates to compare the death risk of immigrants to those of Norwegian-born natives using linked register data on the Norwegian population aged 25–79 during 1990–2015. More than 492,000 deaths occurred in around 4.6 million individuals. All analyses were adjusted for sex, age, calendar time and sociodemographic characteristics.

**Results:**

Immigrants had an 11% survival advantage overall. Those immigrating due to work or education had the lowest death risk, whereas refugees had the highest death risk (albeit lower than that of natives). Death risks increased markedly with length of stay, and were most pronounced for those having spent more than 40% of their lives in Norway. Net of reason for migration, only minor differences were observed depending on Human Development Index characteristics of sending countries.

**Conclusion:**

Independent of reason for migration and characteristics of sending countries, those who immigrate to Norway in adulthood appear to be particularly healthy. The higher death risk associated with prolonged lengths of stay suggests that disadvantageous ‘acculturation’ or stress factors related to the post-migration period may play a role in the long run. The health and welfare of long-term immigrants thus warrants further research.

**Electronic supplementary material:**

The online version of this article (10.1186/s12889-018-5435-4) contains supplementary material, which is available to authorized users.

## Background

Migration has increased over the past decades, and a substantial number of people are currently residing outside their birth country. The health and welfare of migrants are thus relevant for health and welfare policies in host countries. Findings in this area are currently conflicting, in part because migrants comprise a heterogenous group in terms of age, sociodemographic background and length of stay. Furthermore, migrants’ motives for relocation are different. As various host populations’ health also vary, it is not surprising that comparisons show contradictory results.

### Theoretical framework

The theoretical framework employed here focus on selection, cultural adaptation, social status and data irregularities as relevant mechanisms. Selection, cultural adaptation, and social status differentials have been shown to explain a pronounced share of the documented differences in mortality between immigrants and host populations [1]. These mechanisms are likely associated with reason for migration, length of stay, age at migration and sending country characteristics. Depending on the direction and strength of these factors, they may explain either a higher or a lower mortality among immigrants.

### Selection in out-migration

Immigrants are commonly not representative of the population they travel from. Since they have had both the ability and opportunity to migrate, they tend to be more resourceful than the average in the sending country [2, 3]. The selection may also be based on health, i.e. that immigrants are either healthier or sicker than the average in the sending country. Today there is most support for positive health selection [4, 5]. As such, one may expect a lower mortality among immigrants because they will be particularly healthy and strong. This is because it is mostly healthy people who choose to move for work or education [6]. On the other hand, there are also some who argue for a negative health selection, which means that sick people moving in hopes of getting better treatment in a new country [7, 8]. In certain settings, refugees may experience health deteriorations because of circumstances before or during the transfer [9–11].

### Selection in return-migration

Some studies indicate that immigrants to some extent choose to return to their homeland when they get sick or old, to die in their country of origin [12]. This is commonly referred to as the ‘salmon hypothesis’. It is hypothesized that this mechanism may work differently in different countries, in part depending on the availability of health care in the host country versus the country of origin [13–16]. It may also be that the likelihood of returning to one’s country of origin depend on one’s ties to that country, measured for instance by proxies such as age at migration and length of stay in the host country.

### Cultural adaptation, integration and acculturation

There is considerable variation in the degree to which immigrants become integrated in the host society. Although Norway fares relatively well regarding immigrant’s opportunities for participation in society compared to other countries as measured by the MIPEX indicator [17], Norwegian studies show that immigrants tend to have more problems than natives when it comes to finding relevant jobs and get proportionally paid [18]. In terms of education, both undergraduate and graduate education are virtually free in Norway, and there are several special arrangements for immigrants, such as the introduction program, but these are used to varying degrees by different immigrant groups [19].

When it comes to immigrants’ health behavior and use of health services several studies indicate that this varies by country of origin, length of stay and degree of integration in the host country, which we elaborate on below. Many health habits that have an impact on mortality, such as smoking, drinking, diet and physical activity have been shown to vary between migrants and host populations [20]. Immigrants will to varying degrees adapt habits in the country they are moving to, and this could increase or decrease their mortality, depending on whether they primarily adapt health-inducing or health-reducing habits.

Siddiqi et al. found that certain group of immigrants do not utilize health care to the same extent or in the same manner as the host population if they suspect something is wrong, both in countries where services are publicly available and where they must be bought in the private market [21]. Several Norwegian register studies have shown that immigrants in Norway use primary and specialized health care services differently and generally less than natives, though with marked variation within immigrant groups [22–25]. On the other side, immigrants in Norway generally consider their health to be poorer than the general population, especially at older ages [26], although these data rely on a self-selected population. However, immigrants with good social and material resources tend to report relatively fewer health problems, and labor and education immigrants have a better health than refugees and family immigrants [27].

### Social status and social causation

Social status is strongly correlated with mortality, and the composition of immigrants and the host population along important dimensions such as education, marital and parental status vary considerably. However, well-established relationships between these characteristics and mortality for the majority population may be slightly different for some immigrant groups [28]. On the other hand, differences between immigrant groups, particularly related to the reasons for migration, may be overshadowed when sociodemographic characteristics are considered. In a previous study from Norway, the direction of the effect of socio-demographic characteristics was relatively similar between immigrants and the general population [29].

Migration in itself is now regarded as a health determinant independent of other socioeconomic factors previous to, during and after migration [1]. Once in the host country, the health of migrants may be negatively affected if they experience problems as a result of being a minority group beyond what conventional sociodemographic characteristics manage to account for. Marmot, Kogevinas and Elston refer to this as ‘social causation’ [30]. As exemplified above, highly educated immigrants in Norway earn on average less than the general population with similar education, because they tend to up in jobs that are less relevant 18].

### Omissions and inconsistencies in the data

Data irregularities are typical in studies of immigrants. In an international context, Norwegian registers are generally considered to be of high quality. Nevertheless, there are inconsistencies in the registrations of emigration, which in turn may result in ‘statistical immortality’ [5]. This issue is further expanded on in the *Limitation* section.

### Previous empirical research

Research on mortality differences according to *reasons* for migration is scarce. This might be attributed to lack of data as many countries lack registration of reasons for migration. Norway has collected such data from 1990 onwards. Studies from Sweden and Denmark show pronounced differences in mortality between labor and education migrants compared to refugee or family migrants [9, 10]. In Sweden and Denmark, immigrants have lower overall mortality compared to the Swedish/Danish-born [10, 31]. However, refugees are an exception and in Sweden they have the same mortality as the Swedish-born whereas in Denmark it is still lower than that of Danish-born but higher compared to other migration groups [10]. Mortality among refugees due to cardiovascular diseases is also higher compared to Danish-born [32].

Studies on *length* of stay show conflicting results. Several studies show that the health and mortality of immigrants is comparable to the host population the longer immigrants live in the destination country as health worsens and mortality rises [33–37]. Other studies do not find any consistent patterns associated with length of stay [4, 38, 39]. Some studies examine *age* at migration. A lower mortality is observed among immigrants to the United States (US) who migrate after age 24, regardless of length of stay [39]. The study also shows that those who migrate before the age of 18 have similar mortality as the majority population, and thus length of stay play a minor role. Another US study finds lower mortality among immigrants who immigrated after age 50, compared to those migrating during childhood, adolescence or early adulthood [40]. Studies from Canada concur that age at migration plays an important role [4].

Many studies categorize immigrants according to individual countries of origin and compare mortality without focusing explicitly on cross-national similarities or dissimilarities. A few notable exceptions exist: Gadd et al. compare all-cause mortality between immigrants in Sweden and in their country of origin and observe that the mortality is lower for immigrants in Sweden than in their respective birth countries [41]. Rafnsson et al. observe that immigrants’ cardiovascular mortality varies both by geographical region and country of birth within several EU countries, and that it may be either higher or lower than that of the host population [42]. The same patterns are also observed for all-cause mortality [43].

The overall mortality of immigrants in Norway is lower than that of the general population [29]. In this paper, we focus explicitly on the impact of *reason for m*igration, *length* of stay, and *characteristics of sending countries* as measured by the Human Development Index (HDI). Knowledge of various immigrant groups’ mortality can identify risk groups and pinpoint possible areas for public health interventions and integration efforts where policy changes and/or targeted measures may contribute to a better health for immigrants in Norway and similar countries. Such knowledge can also provide important background information for analyses attempting to study the various immigrant groups’ contribution to value creation in society (such as employment), but also their use of resources in relation to the withdrawal of various public benefits (such as pensions) and health care. As immigration is expected to continue to rise, such knowledge will be of increasing relevance in the future.

### Norway’s immigration history

In 1970, immigrants comprised less than 2% of the population in Norway. Even though Norwegian immigration policies were relatively liberal after World War II, immigrants comprised a minor proportion and came mostly from the other Nordic countries. In 1950, refugees from Eastern Europe began to migrate to Norway. Towards the end of the 1960s, also migrant workers from other parts of the world came to Norway. In 1975, there was a labor immigration freeze, and after this the immigrants who came were primarily refugees from Asia, Africa, South America and Eastern Europe. After the EU enlargement from 2004 onwards, there was a sharp increase in labor migration, especially from Poland and Lithuania. Over the last ten years there has been a steady influx of labor migrants from Eastern Europe and refugees and family migrants from low income countries. At present, the distribution among resident immigrants with a known reason for migration is 39% family, 33% labor, 22% refugee status, and 5% education [19]. Today there is a pronounced (13%) and growing immigrant population in Norway [19]. Immigrants make up a very heterogeneous group as to where they come from, and why they come to Norway. Furthermore, there are important differences in their labor market attachment, education, marital status and health. Since Norway has a relatively short history of immigration and immigrants on average are slightly younger than the host population of a country, it is only in recent years that immigrants’ mortality in Norway may be reliably assessed.

## Methods

Registry data from Statistics Norway were compiled for immigrants and Norwegian-born persons with two Norwegian-born parents (hereafter referred to as natives), age 25–79 years, registered as residents in Norway in (parts of) the period 1990 to 2015 (*N* = 4.6 million). The number of immigrants was 808,534. Yearly observations for each subject were constructed (*N* = 75 million). The average follow-up was 16.3 years: 17.8 years for the natives and 8.9 years for immigrants. The probability of all-cause death was analyzed using discrete-time models with time-varying and time-invariant covariates. Interaction terms between relevant immigrant characteristics were added to assess effect modification. The statistical significance level was set at 5%.

Statistics Norway defines immigrants as persons born abroad to two foreign-born parents and four foreign-born grandparents [19]. As such, immigrants have at some point immigrated to Norway. We included the time independent immigrant characteristics *reason for migration* (7 groups), *HDI of sending countries* (6 groups), and *age at migration* (8 groups), as well as the time-dependent characteristic *length of stay* (6 groups). As a robustness check because of the inherent relationship between age at migration and length of stay, we also looked at the *share of life* spent in Norway (4 groups).

Covariates that have been shown to vary with both immigrant characteristics and mortality were included in all models. Education was categorized as higher education (college/university) versus education limited upwards to high school. Annual changes were allowed for calendar period (1990–1994, 1995–1999, 2000–04, 2005–09 and 2010–15), five-year age groups (25–29, ..., 75–79 years), parenthood (yes / no) and marital status (never married, married, surviving spouse and divorced/separated).

A total of 6 different models were set up: 1) Immigrant vs. native; 2) Reason for migration; 3) Length of stay in Norway; 4) Age at migration; 5) Share of life spent in Norway; and 6) Sending countries grouped according to the most recent United Nations’ measure of HDI (http://hdr.undp.org/en/composite/HDI).

Initially, all models were set up separately for men and women, since it is well known that male mortality is higher than female mortality. However, as it appeared that there were minimal differences in the relative death risks by sex and immigrant characteristics (see Table A1, Additional file 1), only combined estimates are presented below.

## Results

A total of around 492,000 deaths were observed: 470742 among the natives and 21,429 among the immigrants. Because immigrants on average are younger than the natives, they constituted 10% of the subjects, 18% of the observations but only 4% of the deaths. As expected, there were more deaths among men (61%) than among women, but the sex distribution was the same for immigrants and natives.

### Descriptive statistics on immigrants

Reason for migration was available for 77% of the non-Nordic immigrants in the sample, and among these refugee, family, labor and education accounted for 27, 25, 20 and 4% of the reasons, respectively. ‘Other reasons’ were quoted for 0.3% whereas the remaining 23% had an ‘unknown reason’ for immigration and was comprised almost exclusively (> 99%) of immigrants who had arrived in Norway prior to 1990. Family immigrants are a heterogenous group, and in some subanalyses they were thus divided into two groups: Family immigrants to refugees (22%) and family immigrants to others (the remaining).

Table 1 portrays the distribution of immigrant characteristics. In terms of length of stay, immigrants who had resided 7–15 years in Norway comprised the largest group (26%), followed by immigrants with 16–30 years of residence (23%). Around 20% had resided < 3 years and 3–6 years, respectively. Around 10% had lived more than 30 years in Norway.

**Table 1:** Descriptive statistics for various immigrant characteristics^a^

	Deaths (21429)		Person-years (7.2 mill)	
	Number	Percent	Number	Percent
Reason for migration				
Refugee	3568	16.7	1.6 mill	22.0
Family	1527	7.1	1.5 mill	20.5
Labor	846	3.9	1.2 mill	16.4
Education	81	0.4	252,181	3.5
Other	80	0.4	19,185	0.3
Nordic immigrants	7431	34.7	1.3 mill	18.6
Unknown	7896	36.8	1.4 mill	18.9
Length of stay				
< 3 years	1294	6.0	1.5 mill	21.4
3–6 years	1624	7.6	1.4 mill	20.1
7–15 years	3220	15.0	1.9 mill	26.0
16–30 years	5803	27.1	1.6 mill	22.5
31–45 years	7005	32.7	604,610	8.4
> 46 years	2483	11.6	117,609	1.6
Age at immigration				
Age < 3	123	0.6	52,955	0.7
Age 3–6	135	0.6	91,905	1.3
Age 7–15	604	2.8	337,066	4.7
Age 16–18	601	2.8	274,292	3.8
Age 19–30	8168	38.1	3.8 mill	53.3
Age 31–45	7859	36.7	2.1 mill	29.7
Age 46–60	2738	12.8	403,650	5.6
Age > 60	1201	5.6	61,787	0.9
Human Development Index^b^				
EU countries	5025	23.4	1.9 mill	26.4
Other European countries	2109	9.8	795,466	11.0
Low HDI (< 55)	1865	8.7	882,353	12.2
Medium HDI (55–74)	2365	11.0	1.4 mill	18.7
High HDI (≥ 75)	2634	12.3	941,577	13.1

^a^Descriptive statistics (numbers and percentages) for deaths and person-years by immigrant characteristics. The distributions for socioeconomic covariates are shown in the Additional file 1, Table A2

^b^HDI categorizations were applied to countries outside Europe, according to the United Nations’ classification. The most recent figures available were employed. The Nordic immigrants were categorized separately and are portrayed in the reason for migration category above. As such, the percentages do not add up to a 100 for this category

The majority (53%) of immigrants migrated in young adulthood (age 19–30), followed by adults age 31–45 (30%). Only 7% immigrated as children (age < 16), and 4% as adolescents (age 16–18). The remaining 6% migrated after age 45.

Immigrants from 225 countries are represented in our sample. Just under one-fifth (19%) came from other Nordic countries, 26% from other EU countries and 11% from the remaining European countries. Countries outside Europe were categorized according to HDI: 13% represented high, 19% medium, and 12% low HDI countries.

### Multivariate analyses

**Fig. 1:**
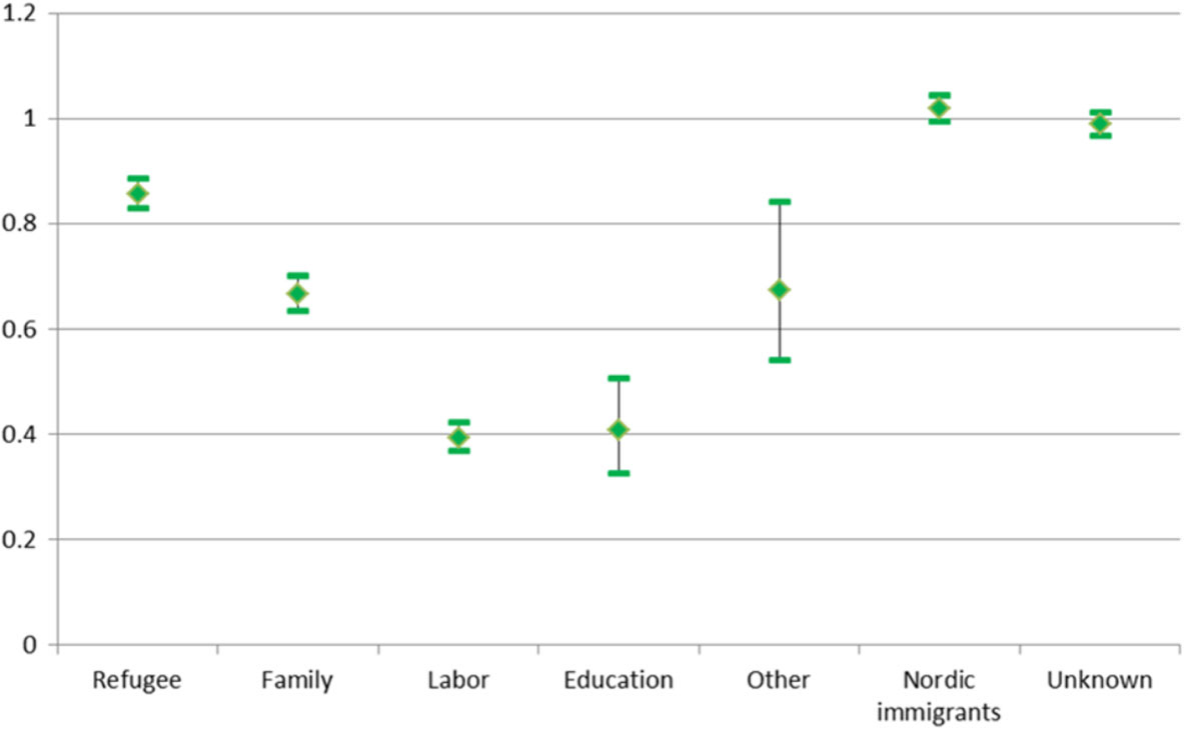
Relative risk of death for immigrants by reason for migration compared to natives. Odds ratios (OR) and 95% confidence intervals, adjusted for sex, age group, calendar period, education, parental and marital status. OR = 1 for natives

Figure 1 shows fully adjusted odds ratios (OR) and 95% confidence intervals (CI) for death by reason for migration relative to that of natives. Nordic immigrants and immigrants with unknown reasons for immigration had the same death risk as natives. For all the remaining groups mortality was lower than that of natives; 14% lower for the refugees and around one-third lower for family immigrants. When we split this group into family immigrants to refugees and others (not shown), there was no significant difference in the death risk of those who joined a refugee (OR 0.69) or a non-refugee family member (OR 0.66). Education and labor immigrants had the lowest overall death risk, around 60% lower than that of natives. Overall and sex-specific distributions and estimates are shown in the Additional file 1 (Table A1), along with corresponding figures for the covariates (Additional file 1: Table A2).

**Fig. 2:**
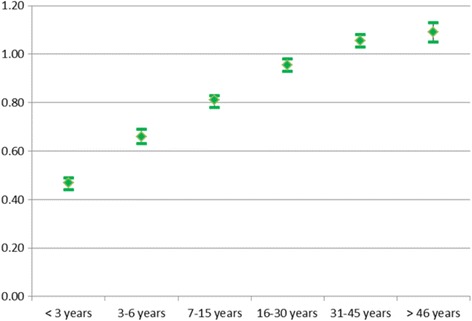
Relative risk of death for immigrants by length of stay in Norway compared to natives. Odds ratios (OR) and 95% confidence intervals, adjusted for sex, age group, calendar period, education, parental and marital status. OR = 1 for natives

Figure 2 portrays the impact of length of stay in Norway, adjusted for age, and shows that the risk of death appears to increase approximately linearly with increasing lengths of stay, up to around 30 years. Compared to natives, the risk of death was significantly lower for immigrants who had lived in Norway less than 30 years. It was halved for immigrants with the very shortest length of stay, while it was around 10% higher for immigrants who had lived in Norway for more than 30 years.

**Fig. 3:**
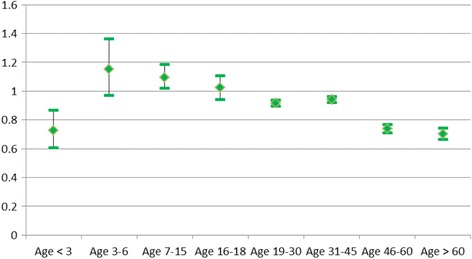
Relative risk of death for immigrants by age at migration compared to natives. Odds ratios (OR) and confidence intervals, adjusted for sex, age group, calendar period, education, parental and marital status. OR = 1 for natives

Figure 3 shows the impact of age at migration. The risk of death is lowest for those who arrived in Norway as fairly newborns (age 0–2) or mature adults (age 46+). These immigrants had 20–25% lower death risks than those of natives. For immigrants who arrived at ages 3–6 and 16–18, the death risk was like that of natives, while it was 10% higher for those who arrived at age 7–15.

**Fig. 4:**
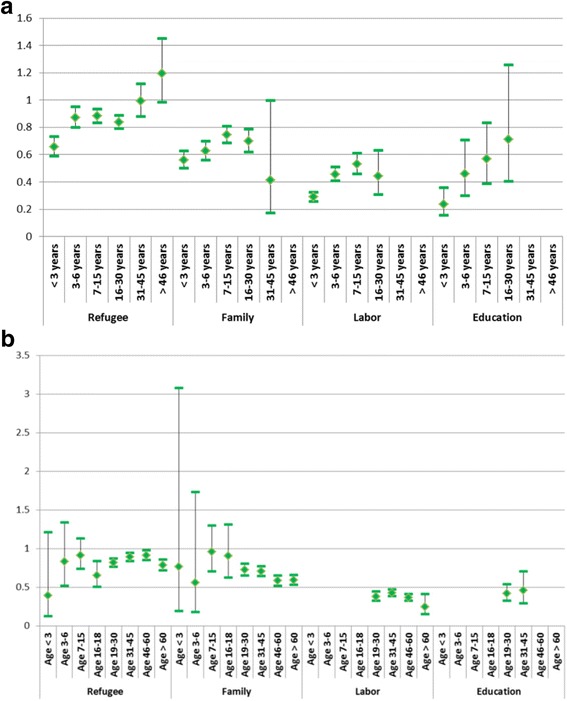
**a** Relative risk of death by reason for migration and length of stay. **b** Relative risk of death by reason for migration and age at migration. Odds ratios (OR) and 95% confidence intervals for immigrants who came as refugees, for family reunification, labor or education, by **a**) length of stay and **b**) age at migration, adjusted for sex, age group, time period, education, parental and marital status. OR = 1 for natives

The reasons why people choose to migrate in the first place, and choose Norway specifically, have changed significantly over time, which in turn affects the composition of the immigrant population in Norway. Consequently, there is interdependence between age at migration and length of stay, and reason for migration. Figure 4a and b thus portray the impact of age at migration (a) and length of stay (b) for immigrants by their reason for migration to Norway. The respective estimates and confidence levels are supplied in Table A3 (Additional file 1).

The impact of length of stay differs for refugees and education immigrants compared to family and labor immigrants (Fig. 4a). While the risk of death increases almost linearly with increasing length of stay for the former groups, there is a tendency to lower death risks with increasing lengths of stay for the latter groups. All migrants, regardless of the reason for migration, have a significantly lower death risk than that of natives if they have spent 15 years or less in Norway. Only long-term refugees have a significantly higher death risk.

Also, when we take age at migration into account, immigrants who migrate for education and labor have, by far, the lowest death risks. Their specific age at migration does not, however, appear to matter (Fig. 4b). The death risk of refugees who migrate during childhood (before age 16) is similar to that of natives. After that, their risk of death is below that of natives. The point estimates suggest that the risk of death increases almost linearly with increasing age at migration, up to age 60. However, the confidence intervals overlap. Also family immigrants who arrive during childhood (before age 19) have a similar death risk to that of natives. After this age, they have a lower death risk, decreasing with increasing age at migration. Also here, the confidence intervals largely overlap.

### Interdependence between characteristics of sending countries and reason for migration

Multivariate results of the characteristics of sending countries are shown in Table A1 (Additional file 1). In short, there is less variation in mortality across sending countries as measured by HDI than across other immigrant characteristics (from OR 0.75 for medium HDI countries to OR 0.99 for low HDI countries). The impact of the HDI characteristics of sending countries does not appear to be linear.

**Fig. 5:**
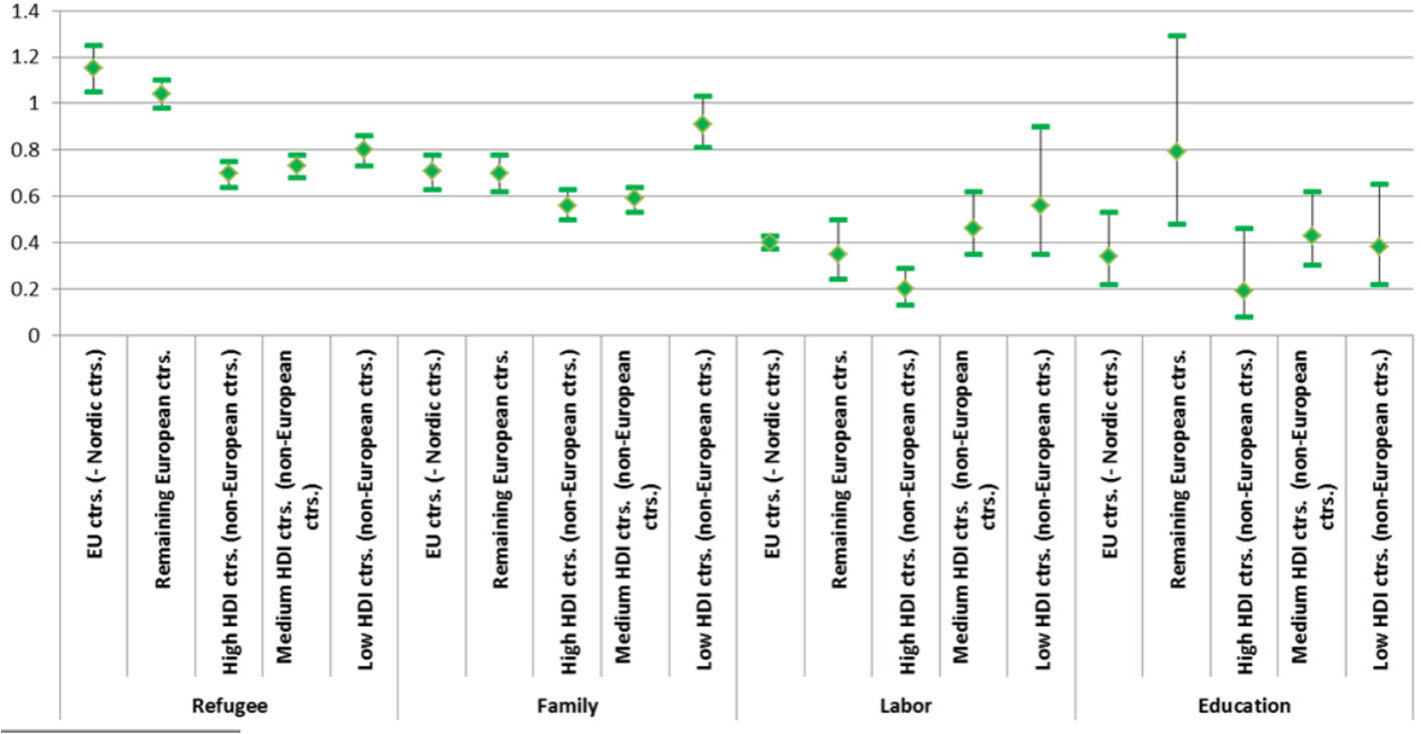
Relative risk of death by reason for migration and HDI of country of origin. Odds ratios (OR) and 95% confidence intervals for immigrants who came as refugees, for family reunification, labor or education, by Human Development Index (HDI) of country group of origin, adjusted for sex, age group, calendar period, education, parental and marital status. OR = 1 for natives

Figure 5 portrays estimates for the interaction between sending country characteristics in terms of HDI and reason for migration. Refugees from the EU countries comprise the only group with a statistically significant higher death risk than natives. When we examine this in more detail, we find that refugees from the former Soviet Union and other Eastern-European countries drive this result. Refugees from non-EU European countries have a similar death risk to that of natives, as is also the case for family immigrants from low HDI countries and education immigrants from non-EU European countries. All other groups have a statistically significant lower death risk than natives.

## Discussion

Immigrants in Norway have lower all-cause mortality than natives, but their mortality differs depending on reason for migration, length of stay, and age at migration. As such, our findings are consistent with much international research. However, there are also some differences. In Norway, female and male immigrants have roughly the same relative survival advantage, in contrast to findings from for instance the US [20] and Denmark [10]. As the differences between male and female life expectancy in the host populations are roughly similar (4–5 years at birth) in the US, Denmark and Norway, and the ratios of male to female immigrants roughly equal (about 50%), we are unable to explain why our results differ from that of others. Consequently, sex differences are not discussed further.

### Selection in out-migration

As stated in the theoretical framework, immigrants are not a representative sample of their countries of origin. They often tend to be above average resourceful [2, 3], which may explain the relatively weak impact of sending countries’ HDI on death risks. In line with this, our results suggest that *reason* for migration appears to matter more. The difference in mortality among immigrants from various areas of origin is most pronounced for refugees and family migrants – while there is more consistency in mortality for labor and education immigrants across areas and individual countries.

Health selection, that migrants are either healthier or sicker than the average in the sending country, is one explanation. Our findings support an overall ‘healthy migrant’ selection, in line with results of others [4, 5]. Healthy people will move for work or education [6], as is supported by our results which show that immigrants who come to Norway for education and/or labor have the lowest mortality, while refugees and family immigrants to refugees had a higher mortality, though lower than native Norwegians. Our findings align well with a recent Norwegian study on immigrants’ health, finding that labor and education immigrants have better health than refugees and family immigrants [27], and studies which find that the same is true for primary health care utilization [22] and hospitalizations [23]. Since the Nordic countries have comparable welfare systems related to health, education and income, we find it reasonable that the Nordic immigrants in Norway have similar mortality as native Norwegians.

On the other hand, certain groups of immigrants may have poorer health and higher mortality than the general population [33, 38]. This has been explained by stress, trauma and other adverse health exposures related to the migration process, such as changes in social status [44] or conditions related to ‘forced’ migration, often characteristic for the situation of refugees [9–11]. Last, some sick people will move in hopes of getting better treatment in a new country [7, 8]. However, regardless of reason for migration, no evidence of worse health was found in the present study, and our results thus suggest that the ‘healthy migrant’ selection undermines any such effect, particularly in the short run.

In both Denmark and Sweden, immigrants generally have the same relatively low mortality as immigrants in Norway [10, 41]. However, the risk of dying varies between different immigrant groups, and unlike what we find in Norway, some immigrant groups in the other Nordic countries have equal or higher mortality [10, 33].

Immigration to Norway has shifted considerably over time. This applies both for reason for migration, timing, and the countries (areas) people migrate from. This makes it difficult to distinguish the importance of reason for migration, age at migration, proportion of life spent in Norway and/or place of origin. When we compare findings across these factors, we find some differences: For refugees, mortality is lowest for those who migrated at a young age, before it increases with increasing lengths of stay. The opposite pattern is seen for family immigrants: Mortality is lowest for those who migrate at older ages and then drops with increasing lengths of stay. As such, the positive selection of adult immigrants may not fully extend to include their children or younger family members. It may thus be relevant to take the reason for migration or underlying factors that are associated with the migration into consideration in the analysis of the importance of length of stay and age at migration, especially as immigrants gain experience in a host country. For immigrants with relatively short lengths of stay in Norway, all groups, regardless of the reason for migration, have significantly lower mortality than natives. This may suggest an initial positive health selection [10].

### Selection in return-migration

According to the theoretical framework, immigrants may choose to return to their countries of origin when they get sick or old, to die in their country of origin [12]. In the United States, privatized health care might be a driver for this hypothesis [13, 14]. In the Nordic countries, virtually free public health services are available for all. As the risk of death increases with length of stay, our findings do not support selective re-migration of sick individuals, in line with results from for instance Denmark and the Netherlands [15, 16].

### Integration, cultural adaption and health

There is considerable variation in the extent to which immigrants become integrated in the host society [17], including the extent to which they adapt lifestyle factors that promote or reduce their health [21, 45]. As we rely on mortality data as a proxy for health, we are unable to shed light on whether Norwegian immigrants’ lower use of health services reflect a better health or an underuse of health services. Below we attempt to discuss mortality patterns by length of stay and age of migration to hypothesize whether the lower mortality we observe among immigrants may be a result of their health being better than that of the host population.

### Length of stay and age at migration

We find that the mortality of immigrants increases with prolonged lengths of stay. This conflicts with some findings [4, 38, 39], but is concordant with the hypothesis of adverse adaptation and consistent with findings from others [36, 37]. It is also consistent with the impact of migration as an independent determinant of health and with a ‘social causation’ interpretation, i.e. that immigrant status interacts with sociodemographic disadvantages as conventionally measured, and thus increases mortality [28]. Immigrants with long lengths of stay were pioneers when they arrived, and any positive health selection effects may over time have been offset by long exposure to Norwegian societal structures, habits and risk factors.

Immigrants who arrive in Norway at very young ages have lower mortality than other groups of immigrants. It may be argued that immigrants who arrive in Norway in early childhood will be less affected by *being* immigrants [46]. However, immigration during childhood and adolescence might be detrimental for health, since they show similar mortality levels as natives. Another possible explanation is that the selection bias during infancy is different depending on the age of the child, with the youngest being more susceptible to death in their home countries or during migration. In accordance to our findings, lower mortality was also observed among immigrants in the US and France who arrived at higher ages, regardless of duration of residence [13, 39, 40, 44], in line with the hypothesis of positive health selection [4].

The share of life spent in Norway attempts to simultaneously measure both length of stay and age at migration. Our findings show that mortality is low for immigrants who have lived only a small share of their lives in Norway, before it rises markedly, thus supporting the hypotheses of positive health selection, unfortunate social adaptation, as well as ‘social causation’ and allostatic load, or the health burden of chronic stress, related to migration [47].

### Limitations and future research needs

Although this is a national study with long follow-up and relatively detailed and complete data, several limitations exist: Firstly, registrations of immigrants’ emigration are less complete than for natives. This is problematic because immigrants have greater emigration rates than the majority: In 2011, around 70% of all emigrations concerned immigrants [48]. Furthermore, emigration probabilities depend on the reason for migration, length of stay and age at migration. We know that labor and education immigrants often spend only a short period in Norway. An alternative explanation for positive health selection for the low mortality we find for these groups may thus be missed emigration registrations. In our calculations of risk of death according to these characteristics, we did not detect evidence for consistent misclassifications in a specific direction, and as the risk of death increased with increasing length of stay, we conclude that possible missed emigration registrations are unlikely to drive our results. This is of critical importance since consistent errors in emigration registrations would make immigrants ‘immortal’ in a statistical sense [5], causing immigrants’ mortality to be incorrectly estimated too low.

Secondly, the immigrants included are relatively young because of Norway’s relatively short history of immigration. As such, the deaths we use in the analysis are relatively unevenly distributed for the characteristics under investigation resulting in some unstable estimates. As further research accumulates, our findings may be rebutted.

Thirdly, information on cause of death would have given us a better indication of health disparities, but this information is unfortunately not available and due to the young age of the immigrant population the numbers in the respective subgroups would be small.

Lastly, and most importantly, we lack information on health. Not all health differences translate into mortality differences. Furthermore, some mortality differences (albeit rare) may not relate only to health, such as for instance work accidents. As such, a more proximate measure of health could have given us more policy relevant information. This pertains both to immigrants’ health prior to arriving in Norway, and their health trajectories once in Norway. In general, information on immigrants’ health during their life course in Norway is only available from cross-sectional surveys based on self-selected samples. Knowledge of immigrants’ health prior to arrival in Norway is scarce and mostly anecdotal.

Norway and the other Nordic countries are welfare states with affordable and available health care and income security, resulting in less inequality across various areas of health than in many other countries. However, as we find that the risk of death is low initially, before it increases substantially with increasing length of stay, welfare policies may not successfully benefit immigrants in the long run. Unfortunately, we did not have access to the characteristics of immigrants before they leave their country of origin [2]. However, our results of low mortality shortly after arrival may support the ‘healthy migrant’ hypothesis, perhaps suggesting that the health of migrants prior to migration may be better than the health of the general population in both the sending and the receiving country. On a similar note, we used the most recent United Nations’ HDI measure available for each country, irrespective of the migrants’ year of emigration. It would likely have been more optimal to use the HDI at migrants’ time of emigration from their country of origin. Unfortunately, this date is not available in our data. However, since the majority of the HDI measures have been relatively stable from 1990 onwards for sending countries of the largest immigrant groups in Norway, we believe it is unlikely that a different coding would majorly impact on our results.

Whether our findings may be generalized to countries with dissimilar welfare systems, especially health care systems, remains to be examined, and comparative research in this area is currently largely lacking [49, 50].

## Conclusion

Reason for migration appears to be an important indicator for later mortality, even when one takes sociodemographic characteristics and characteristics of sending countries into account. The low mortality of recent immigrants may suggest that it is the healthiest immigrants who move to Norway in adulthood, even when we account for reason for migration. At the same time, mortality increases with longer lengths of stay and/or shares of lives spent in Norway, perhaps indicating that immigrants in the long-term make adverse health adaptations that have a negative impact on their mortality. It may therefore seem that the various mechanisms that have been discussed here work simultaneously and affect immigrant mortality in different directions. Our study supports the need for research on immigrants’ health and welfare from arrival in a host country, and over the life course as they get older and have more experience in their new host country.

## Additional file

Additional file 1: Tables A1-A3. (PDF 182 kb)

## Abbreviations

CI: Confidence interval
EU: European Union
HDI: Human Development Index
OR: Odds ratio
US: United States of America
