# Supplementary material for: An observational study of immigrant mortality differences in Norway by reason for migration, length of stay and characteristics of sending countries
Source: BMC Public Health. 2018 Apr 17;18:508. doi: 10.1186/s12889-018-5435-4 (PMC5905163; doi:10.1186/s12889-018-5435-4)
Supplement: Supplementary file 1 — Tables A1-A3. (PDF 182 kb) [file 12889_2018_5435_MOESM1_ESM.pdf]

Table A1. Descriptive statistics and relative risk of death by various immigrant characteristics.[a]

	Both sexes combined				Men		Women	
	**Deaths**	**Pyrs**[b]	**OR**[c]	**CI**[d]	**OR**	**CI**	**OR**	**CI**
Natives	470 742	67.8 mill	1	ref	1	ref	1	ref
All immigrants[d]	21 429	7 210 975	0.89	0.88-0.90	0.89	0.87-0.90	0.91	0.89-0.93
Reason for migration								
Refugee	3 568	1 584 574	0.86	0.83-0.88	0.89	0.85-0.92	0.82	0.77-0.87
Family	1 527	1 477 467	0.67	0.63-0.70	0.65	0.60-0.70	0.63	0.60-0.67
Labor	846	1 179 253	0.39	0.37-0.42	0.42	0.39-0.45	0.40	0.34-0.48
Education	81	252 181	0.41	0.33-0.50	0.35	0.26-0.47	0.49	0.36-0.66
Other	80	19 185	0.68	0.54-0.84	0.67	0.50-0.87	0.69	0.48-0.99
Nordic immigrants	7 431	1 338 349	1.02	0.99-1.04	1.01	0.98-1.04	1.05	1.01-1.08
Unknown	7 896	1 359 966	0.99	0.97-1.01	0.98	0.95-1.01	1.02	0.99-1.06
Length of stay								
< 3 years	1 294	1 541 494	0.47	0.44-0.49	0.45	0.42-0.48	0.52	0.48-0.57
3-6 years	1 624	1 447 715	0.66	0.63-0.69	0.64	0.60-0.67	0.73	0.68-0.79
7-15 years	3 220	1 874 590	0.81	0.78-0.83	0.81	0.77-0.84	0.81	0.77-0.86
16-30 years	5 803	1 624 957	0.95	0.93-0.98	0.96	0.93-0.99	0.95	0.91-0.99
31-45 years	7 005	604 610	1.06	1.03-1.08	1.07	1.04-1.10	1.05	1.01-1.08
> 46 years	2 483	117 609	1.09	1.05-1.13	1.13	1.07-1.19	1.06	1.00-1.12
Age at immigration								
Age < 3	123	52 955	0.73	0.61-0.87	0.85	0.68-1.06	0.61	0.46-0.80
Age 3-6	135	91 905	1.15	0.97-1.36	1.18	0.96-1.44	1.12	0.84-1.50

Age 7-15	604	337 066	1.10	1.02-1.18	1.06	0.96-1.17	1.17	1.03-1.32
Age 16-18	601	274 292	1.02	0.94-1.11	1.06	0.95-1.16	0.98	0.86-1.11
Age 19-30	8 168	3 845 669	0.92	0.90-0.94	0.92	0.89-0.94	0.94	0.90-0.96
Age 31-45	7 859	2 143 651	0.94	0.92-0.96	0.93	0.90-0.95	0.99	0.96-1.03
Age 46-60	2 738	403 650	0.74	0.71-0.76	0.74	0.70-0.77	0.76	0.71-0.80
Age > 60	1 201	61 787	0.70	0.66-0.74	0.67	0.62-0.73	0.73	0.67-0.78
Share of life spent in Norway								
< 20 percent	4 320	3 165 991	0.62	0.60-0.64	0.61	0.58-0.63	0.67	0.64-0.70
20-39 percent	4 265	1 868 919	0.87	0.84-0.90	0.86	0.83-0.89	0.88	0.84-0.92
40-60 percent	8 114	1 463 281	1.04	1.02-1.06	1.04	1.01-1.06	1.07	1.03-1.10
> 60 percent	4 730	712 784	1.05	1.02-1.08	1.11	1.06-1.15	1.01	0.96-1.06
Human Development Index[e]								
EU countries	5 025	1 901 381	0.83	0.81-0.85	0.8	0.77-0.82	0.94	0.91-0.98
Remaining European countries	2 109	795 466	0.96	0.92-1.01	1.02	0.97-1.08	0.87	0.81-0.93
Low HDI (< 55)	1 865	882 353	0.99	0.95-1.04	1.01	0.96-1.07	0.98	0.91-1.07
Medium HDI (55-74)	2 365	1 351 849	0.75	0.72-0.78	0.77	0.73-0.80	0.72	0.68-0.77
High HDI (≥ 75)	2 634	941 577	0.80	0.77-0.83	0.79	0.75-0.83	0.84	0.79-0.88

[a]This table shows estimates from 6 different models for both sexes combined, and separately for each sex. Covariates include sex (in combined models), age group, calendar period, education, parenthood and marital status. The estimates and the categorizations of covariates are portrayed in Table A2. [b]Person-years. [c]Odds ratio. [d]95% confidence interval. [e]HDI categorizations were applied to countries outside Europe, according to the United Nations' classification for the most recent year available. The Nordic immigrants were categorized separately but as they are portrayed in the reason for migration analysis above, they are not shown here.

Table A2. Descriptive statistics and relative risk of death for covariates.[a]

	Deaths	Pyrs[b]	OR[c]	CI[d]
Total	492 191	75.0 mill	N/A	N/A
Women	193 698	37.4 mill	1	ref
Men	298 473	37.6 mill	1.94	1.93-1.95
Running age				
25-29 years	4 914	8.0 mill	1	ref
30-34 years	5 844	8.3 mill	1.25	1.20-1.30
35-39 years	7 665	8.5 mill	1.67	1.61-1.73
40-44 years	10 967	8.4 mill	2.40	2.32-2.48
45-49 years	16 698	8.1 mill	3.73	3.61-3.85
50-54 years	24 754	7.4 mill	6.03	5.84-6.22
55-59 years	35 046	6.6 mill	9.55	9.26-9.85
60-64 years	50 943	5.9 mill	15.53	15.07-16.01
65-69 years	74 336	5.3 mill	25.03	24.29-25.79
70-74 years	107 238	4.6 mill	40.44	39.26-41.66
75-79 years	153 766	3.9 mill	67.97	65.99-70.01
Running calendar period				
1990-1994	114 734	13.3 mill	1	ref
1995-1999	105 909	13.9 mill	0.93	0.93-0.94
2000-2004	92 272	14.2 mill	0.84	0.83-0.85
2005-2009	83 618	14.7 mill	0.76	0.75-0.76
2010-2015	95 638	18.9 mill	0.67	0.66-0.67
Sosiodemographic charactersitcs				

0 children[e]	116 645	12.6 mill	1	ref
1+ children	375 526	62.4 mill	0.73	0.73-0.74
Never married[e]	86 131	21.0 mill	1	ref
Married	248 945	41.4 mill	0.63	0.63-0.64
Widow(er)	85 305	3.8 mill	0.89	0.88-0.90
Divorced/separated	71 790	8.8 mill	1.13	1.12-1.14
Low education[e,f]	378 786	35.4 mill	1	ref
High education	113 385	39.6 mill	0.63	0.63-0.64

[a]This table portrays covariate estimates from the model on reason for migration. [b]Person-years. [c]Odds ratio. [d]95% confidense interval. [e]The reference category includes observations with missing information on this variable. This pertained to very few observations in total, but a more pronounced share among immigrants than among natives. The effect magnitude and the confidence interval for the various immigrant charateristics are virtually similar if the missing observations are included in a separate category or included in the referenec category. [f]Low education is limited upwards to high school.High education refers to any college or university level education.

Table A3. Estimates from combined analyses of reason for migration and a) length of stay and b) age at migration.[a]

	Refugee			Family			Labor			Education		
	**N**	**OR**[b]	**95% CI**[c]	**N**	**OR**	**95% CI**	**N**	**OR**	**95% CI**	**N**	**OR**	**95% CI**
a) Length of stay												
**< 3 years**	234 697	0.65	0.59-0.73	375 989	0.56	0.50-0.62	573 976	0.29	0.26-0.33	106 187	0.23	0.15-0.36
**3-6 years**	297 933	0.86	0.79-0.94	391 679	0.62	0.56-0.70	413 874	0.46	0.41-0.51	68 593	0.47	0.30-0.72
**7-15 years**	567 099	0.88	0.83-0.94	523 041	0.75	0.69-0.81	172 793	0.54	0.46-0.62	64 205	0.58	0.39-0.85
**16-30 years**	447 622	0.85	0.80-0.91	184 302	0.71	0.63-0.80	18 610	0.45	0.32-0.65	13 196	0.73	0.41-1.28
**31-45 years**	32 762	1.01	0.89-1.14	2 342	0.42	0.17-0.99	0	N/A	N/A	0	N/A	N/A
**> 46 years**	4 461	1.22	1.00-1.48	114	N/A	N/A	0	N/A	N/A	0	N/A	N/A
b) Age at migration												
**Age < 3**	9 123	0.41	0.13-1.28	3 421	0.81	0.20-3.26	1	N/A	N/A	0	N/A	N/A
**Age 3-6**	27 724	0.89	0.55-1.43	8 564	0.60	0.19-1.88	6	N/A	N/A	0	N/A	N/A
**Age 7-15**	106 114	0.96	0.77-1.19	63 234	1.03	0.76-1.40	0	N/A	N/A	191	N/A	N/A
**Age 16-18**	84 569	0.68	0.53-0.87	45 974	0.96	0.66-1.40	660	N/A	N/A	6 094	N/A	N/A
**Age 19-30**	766 934	0.84	0.79-0.90	765 011	0.75	0.67-0.84	521 484	0.41	0.35-0.48	212 784	0.44	0.34-0.57
**Age 31-45**	472 316	0.90	0.85-0.96	486 989	0.71	0.65-0.78	530 898	0.43	0.39-0.48	31 358	0.47	0.30-0.72
**Age 46-60**	95 194	0.91	0.85-0.98	81 774	0.58	0.52-0.65	123 095	0.37	0.32-0.41	1 608	N/A	N/A
**Age > 60**	22 600	0.78	0.71-0.85	22 500	0.59	0.53-0.66	3 109	0.25	0.15-0.41	146	N/A	N/A

[a]This Table provides estimates and confidence intervals for Figures 4a and 4b. N/A refers to not applicable, as an estimate could not be calculated. Both models are fully adjusted (see Table A2). [b]Odds ratio. [c]Confidence interval.
